# Effects on *Capsicum annuum* Plants Colonized with *Trichoderma atroviride* P. Karst Strains Genetically Modified in *Taswo1*, a Gene Coding for a Protein with Expansin-like Activity

**DOI:** 10.3390/plants10091919

**Published:** 2021-09-15

**Authors:** Ricardo Sánchez-Cruz, Richa Mehta, Karina Atriztán-Hernández, Olivia Martínez-Villamil, María del Rayo Sánchez-Carbente, Ayixon Sánchez-Reyes, Verónica Lira-Ruan, Carlos Alberto González-Chávez, María Luisa Tabche-Barrera, Roberto Carlos Bárcenas-Rodríguez, Ramón Alberto Batista-García, Alfredo Herrera-Estrella, Edgar Balcázar-López, Jorge Luis Folch-Mallol

**Affiliations:** 1 Centro de Investigación en Biotecnología, Universidad Autónoma del Estado de Morelos, Cuernavaca 62209, Morelos, Mexico; rsan9207@gmail.com (R.S.-C.); richa.bt@gmail.com (R.M.); inatriztan@gmail.com (K.A.-H.); maria.sanchez@uaem.mx (M.d.R.S.-C.); 2 Laboratorio Nacional de Genómica para la Biodiversidad, Cinvestav Campus Guanajuato, Km. 9.6 Libramiento Norte, Carretera Irapuato León, Irapuato 36824, Gto, Mexico; alfredo.herrera@cinvestav.mx; 3 Facultad de Ciencias Biológicas, Universidad Autónoma del Estado de Morelos Cuernavaca, Cuernavaca 62209, Morelos, Mexico; chrysomeliidae@gmail.com; 4 Cátedras CONACyT-Instituto de Biotecnología, Universidad Nacional Autónoma de México, Cuernavaca 62209, Morelos, Mexico; ayixon.sanchez@mail.ibt.unam.mx; 5 Centro de Investigación en Dinámica Celular, Instituto de Investigación en Ciencias Básicas y Aplicadas, Universidad Autónoma del Estado de Morelos, Cuernavaca 62209, Morelos, Mexico; katlira@uaem.mx (V.L.-R.); rabg@uaem.mx (R.A.B.-G.); 6 Instituto de Biotecnología, Universidad Nacional Autónoma de México, Cuernavaca 62209, Morelos, Mexico; carloschavezplantae@gmail.com (C.A.G.-C.); tabche@gmail.com (M.L.T.-B.); 7 Departamento de Ingeniería Bioquímica, Cinvestav Irapuato, Libramiento Norte Carretera Irapuato León Kilómetro 9.6, Carretera Irapuato León, Irapuato 36821, Gto, Mexico; chino_3005@hotmail.com; 8 Centro Universitario de Ciencias Exactas e Ingeniería, Departamento de Farmacobiología, Universidad de Guadalajara, García Barragán # 1451, Guadalajara 44430, Jalisco, Mexico

**Keywords:** *Trichoderma*, phytopathogen resistance, stress tolerance, SWOLLENIN

## Abstract

Here, we analyzed the effects on *Capsicum annuum* plants of *Trichoderma atroviride* P. Karst strains altered in the expression of SWOLLENIN (SWO1), a protein with amorphogenic activity on plant cell wall components. Strains of *T. atroviride* that overexpressed the *Taswo1* gene were constructed as well as deletion mutants. A novel, cheap and accurate method for assessing root colonization was developed. Colonization assays showed that the *Taswo1* overexpressing strains invaded the host root better than the WT, resulting in a stronger plant growth-promoting effect. The expression of plant defense marker genes for both the systemic acquired resistance and induced systemic resistance pathways was enhanced in plants inoculated with *Taswo1* overexpressing strains, while inoculation with deletion mutant strains resulted in a similar level of expression to that observed upon inoculation with the wild-type strain. Response to pathogen infection was also enhanced in the plants inoculated with the *Taswo1* overexpressing strains, and surprisingly, an intermediate level of protection was achieved with the mutant strains. Tolerance to abiotic stresses was also higher in plants inoculated with the *Taswo1* overexpressing strains but was similar in plants inoculated with the wild-type or the mutant strains. Compatible osmolyte production in drought conditions was studied. This study may contribute to improving *Trichoderma* biocontrol and biofertilization abilities.

## 1. Introduction

In recent years, biofertilizer products containing different types of living microorganisms have emerged as important components in integrated nutrient supply systems and hold a great promise to improve the yield and quality of crops [1]. Among the most widely applied biofertilizers are fungi from the genus *Trichoderma* [2].

*Trichoderma* spp. are ubiquitous soil fungi commonly found also in a large range of plant roots. They establish plant symbioses with monocots as well as dicots, and it has been proposed that there is little or no plant specificity [3,4,5]. In the work by Zaidi et al., in [6], it has also been stated that “the mechanisms determining host specificity remain poorly understood”. *Trichoderma* spp. have also been widely studied since they show mycoparasitism against other fungi [7]. The strains that are able to colonize roots in a long-term period penetrate the epidermis and reach some cells below this level [3]. Furthermore, they synthesize and secrete different compounds, which can induce resistance responses in the plant against fungal and bacterial pathogens [5,8], which is also part of their capability as agents for biocontrol. These molecules are known as MAMPs (microbe-associated molecular patterns) and include a variety of different compounds. Among the most studied are flagellin, glycoside hydrolases, expansins, cerato-platanins, chitin, lipopolysaccharides, hydrophobins, and secondary metabolites such as sorbecillinoids, alamethicin, or orsellinic acid [9]. In response to these molecules, plants have been shown to induce two kinds of immunity: systemic acquired resistance (SAR) and induced systemic resistance (ISR). The first is mediated by salicylic acid, while the latter involves jasmonic acid and ethylene. Nevertheless, recent studies have shown that these responses are complex, and there is crosstalk between both (and other) pathways (such as Fe deficiency responses) [10]. Among others, these defense pathways induce the expression of proteins named PR (pathogenesis-related proteins) in the SAR response, or defensins, in the ISR response. PR are usually hydrolytic enzymes such as chitinases, glucanases or peroxidases, etc., that attack the cell wall of the pathogens. Defensins are small proteins that show antimicrobial activity by disrupting the pathogens’ membrane but their role is still controversial, since they have been involved in abiotic stress tolerance and plant developmental processes as well [11]. Moreover, when *Trichoderma* spp. colonize plant roots, they promote plant growth through the control of pathogenic microorganisms in the root neighborhood [12], enhancing nutrient availability [4,13] and/or secreting plant hormones [14,15]. Furthermore, plant cell wall-degrading enzymes, such as cellulases produced by *Trichoderma* spp., have been shown to be important for plant root colonization [16,17]. Another valuable and interesting feature of the colonization of plants by *Trichoderma* spp. is that it also enhances tolerance to abiotic stress [18,19]. The mechanisms that *Trichoderma* induces in plants to cope with several abiotic stresses (drought, salinity, heat, cold, etc.) are mostly related to scavenging reactive oxygen species (ROS) by a series of molecules that include peroxidases, lipoxygenases, superoxide dismutase, catalase, glutathione reductase and polyphenols, among others [5,18,20]. *Trichoderma* also modifies the root architecture and growth through pH changes allowing a better water and nutrient uptake [21]. In addition, accumulation of compatible solutes such as proline in response to drought stress has been reported [22].

*Trichoderma* spp. also secrete proteins with amorphogenic activity on cellulose, among which cerato-platanins are important to colonize the root but also to trigger the defense responses in the plant [23,24,25,26]. In addition to hydrolytic enzymes, some *Trichoderma* spp. secrete a non-hydrolyzing protein named SWOLLENIN that reshuffles the crystalline cellulose fiber without producing detectable amounts of reducing sugars [27]. SWOLLENIN was first characterized in the saprophytic fungus *Trichoderma reesei* [27] and has been described in other *Trichoderma* species and other ascomycetes such as *Aspergilli* and *Penicillium* species [28,29]. It contains a homologous region related to expansins and a carbohydrate-binding module (CBM), and it has been shown to have amorphogenic activity on cellulose fibers. These proteins have been mostly studied for their capacity to enhance the saccharification of cellulose when added previously to the incubation of cellulose with cellulases. The *Aspergillus fumigatus* ortholog SWO1 binds chitin [28] while SWO1 from *Trichoderma pseudokoningi* has a mild hydrolytic activity on xylan and yeast glucans but not in β-1,4 glycosidic bonds [30]. An alternative role for SWOLLENIN in *T. atroviride* P. Karst has been proposed by Reithner et al. [31], since they found a significantly up-regulated expression of *Taswo1* just before contact with different phytopathogenic fungi. This finding suggests that SWOLLENIN could be involved in causing amorphogenesis in the pathogen fungal cell wall during mycoparasitism. Another role explored for SWOLLENIN was described by Brotman et al. [3]; they observed that overexpression of SWOLLENIN in *Trichoderma asperellum* resulted in a remarkable increase in the ability of the fungus to colonize cucumber roots and that a synthetic peptide corresponding to the CBM of this protein on its own was sufficient to stimulate local defense responses “in vitro” and to induce resistance to stress.

The *T. atroviride* genome sequence revealed the presence of a homologous SWO1 gene (MycoCosm ID80187) to that of *T. reesei* [31]. In this work, we used *T. atroviride Taswo1* overexpressing strains or *Taswo1* deletion mutants to study its interaction with two Solanaceae, chili (*Capsicum annuum*) and tomato (*Solanum lycopersicum*) plants. Our results show that plants inoculated with the overexpressing strains produce more biomass, likely because they colonized the roots more extensively than when inoculated with the wild type. Infection of plants by pathogenic fungi was better controlled when inoculated with the overexpressing strains than infection of those inoculated with the wild-type strain or non-inoculated plants. Surprisingly, the mutant strains enhanced pathogen resistance in leaves to levels in between to those inoculated with the overexpressing strains and the wild type. The expression of defense-related genes was also enhanced in plants inoculated with the overexpressing strains as compared with those non-inoculated or inoculated with the wild-type strain. Our contribution in this work involves mostly in vivo assays of adult plant growth, poses a paradox in the classical model of pathogen resistance, hints at some specificity issues about *Trichoderma*-plant interaction, and includes osmolyte analysis upon imposed drought stress.

## 2. Results

### 2.1. Overexpression of Taswo1 of T. atroviride Promotes Growth and Development of C. annuum and S. lycopersicum Plants

To study the impact of overexpression of SWO1 on the plant growth-promoting activity of *T. atroviride* P. Karst, we transformed the *T. atroviride* wild-type (WT) strain IMI206040 with pUE10::*Taswo1*. The pUE10 vector drives expression from the *T. reesei PKI* constitutive promoter and was designed to integrate approximately 1200 bp downstream the *BLU*17 terminator locus at the *T. atroviride* genome where no evident open reading frames (ORFs) were identified [32]. Transformant strains with the empty vector integrated pUE10 in the desired locus and did not show differences in phenotype from the WT strain regarding growth rate, sporulation, and mycoparasitism [32] (Table 1).

Several pUE10:*Taswo1* independent transformant lines and mutant strains were analyzed and characterized regarding their growth rate (Table 1). When analyzed by Southern blot, most of them showed the expected integration event (for example, see Figure S1). Since the transformed *Taswo1* lines showed similar phenotypes, we chose *Taswo1*-28 and *Taswo1*-10 and mutant strains *Taswo1*-6Δ and *Taswo1*-8Δ to study its interaction mainly with *C. annuum* (although another Solanaceae, *Solanum lycopersicum,* was also partially tested, see Supplementary Material). Overexpression of *Taswo1* was verified through RT-PCR for *Taswo1*-28, *Taswo1*-10, *Taswo1*-6Δ, and *Taswo1*-8Δ (Figure S2).

**Figure 1:**
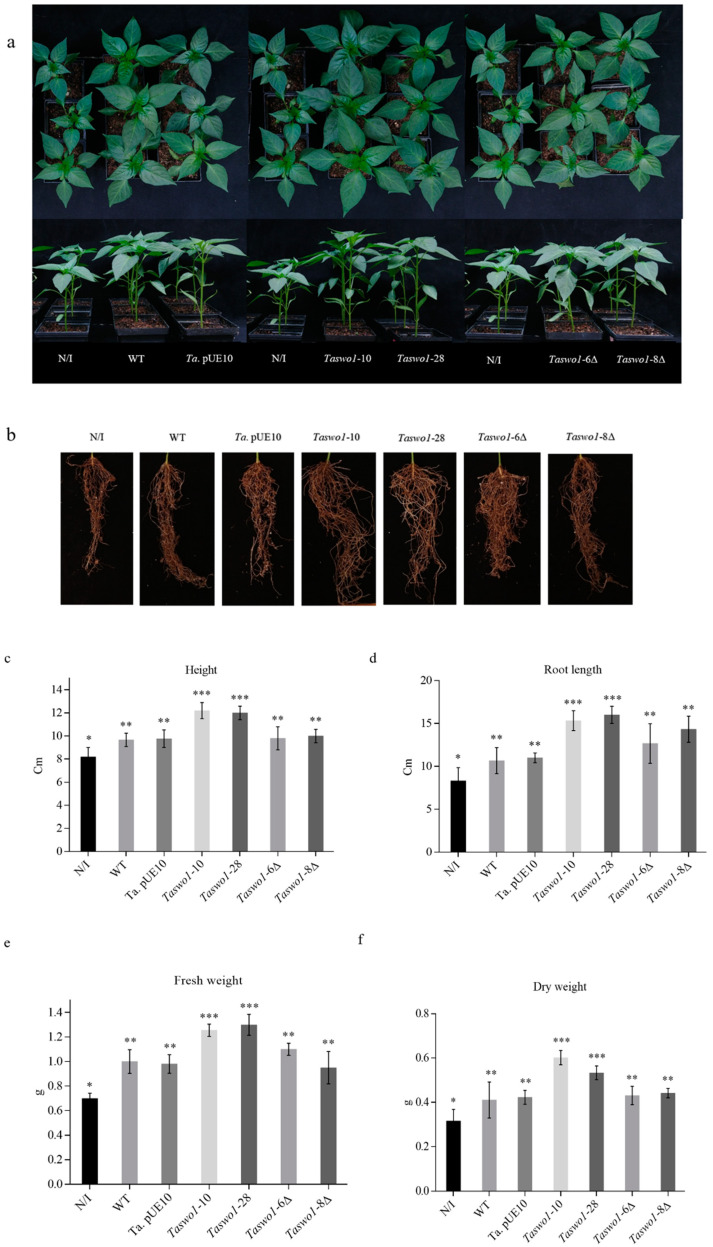
*Trichoderma* overexpressing strains promote plant growth. (**a**) Representative images of *C. annuum* plants treated with the different strains. N/I non-inoculated; (**b**) representative pictures of the root systems of plants inoculated with different strains; (**c**) average height of plants treated with the different strains; (**d**) average length of the root systems of plants with the different *Trichoderma* strains. Panels (**c**,**d**) depict the units in cm; (**e**) fresh and (**f**) dry weights expressed in grams (g) of the whole plants treated with the different *Trichoderma* strains. Experiments were conducted in triplicate. Bars represent standard deviation. Different numbers of asterisks (*) within the panels indicate that the average of each treatment is significantly different (*p* < 0.05, *n* = 3).

To evaluate the effect of overexpression of SWOLLENIN by *Trichoderma* in chili, plants were inoculated with the following strains: WT, empty vector, *Taswo1*-28 or *Taswo1*-10, *Taswo1*-6Δ, and *Taswo1*-8Δ, as described in material and methods. After 30 d of growth, root and shoot growth were determined (Figure 1). Interestingly, inoculation with overexpressing strains *Taswo1*-28 or *Taswo1*-10 strongly stimulated root system growth (Figure 1), increased shoot biomass production (Figure 1), and increased plant height (Figure 1) when compared with the WT or the empty pUE10 inoculated plants, indicating a beneficial effect of the overexpressing on plant growth and development. Non-inoculated plants had shorter shoots and less root system growth, while *Trichoderma* WT-treated plants had longer shoots than the non-inoculated control plants but less than those treated with the overexpressing strains. *Trichoderma*-treated plants had a denser root system. After 30 days, we observed that plant height and leaf area of plants inoculated with *Taswo1*-10 and *Taswo1*-28 had increased almost three-fold that of non-inoculated plants. ANOVA test results (Figure 1c) indicated significant differences in height, fresh weight, and dry weight of the total biomass between plants inoculated with the overexpressing strains or the WT strain, the empty vector transformant, and the control treatments. The means for root length were higher for the overexpressing strains, but statistical analysis showed no significant differences for this feature when compared to the roots of plants inoculated with the other strains (Figure 1d).

*Taswo1* mutants were also tested on *C. annuum* plants. Two independent lines, *Taswo1*-6Δ and *Taswo1*-8Δ, produced the same phenotype in chili plants as the WT *Trichoderma* strain indicating that SWOLLENIN is not essential for plant interaction (Figure 1).

*Phaseolus vulgaris* was also inoculated with the overexpressing strains, the WT, or a non-inoculated control, but no significant growth-promoting phenotype was observed with any of the strains (insignificant changes in root and shoot mass were observed, data not shown). These results indicate that the interaction of this plant with *T. atroviride* has specific components that remain to be further explored.

Since the experiments performed with *P. vulgaris* did not show any significant phenotype differences among the plants inoculated with the different strains (data not shown), we decided to measure plant growth-promoting activity in another plant from the Solanaceae family. *S. lycopersicum* plants showed similar behavior to those experiments performed with chili. Biomass was also enhanced in those plants inoculated with overexpressing *Taswo1*-28. The height of the plants was larger than the controls (Supplementary Figure S3); fresh and dry weight confirmed the larger biomass achieved by plants inoculated with *Taswo1*-28 compared to the controls and showed similar differences in biomass regarding shoot and leaf area and root fresh and dry weights (Supplementary Figure S4).

### 2.2. Root Colonization Assays by the T. atroviride Genetically Modified Strains

**Figure 2:**
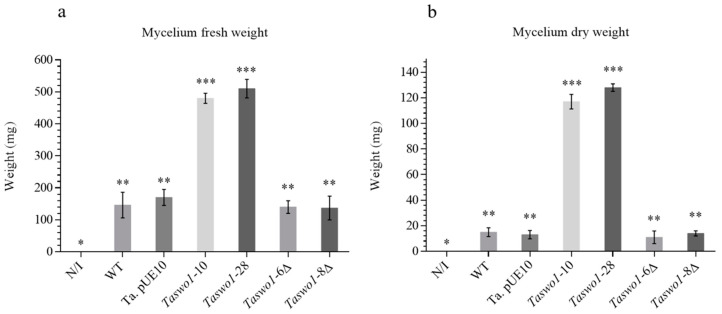
Colonization assay of chili roots by different strains of *Trichoderma atroviride* or in non-inoculated plants. Mycelium growth extracted from roots of non-inoculated plants or inoculated with the WT or with the strain with the empty plasmid *Ta*pUE10, with the overexpressing lines *Taswo1*-10, and *Taswo1*-28 or mutant lines *Taswo1*-6Δ and *Taswo1*-8Δ ANOVA variance test of fresh (**a**) and dry (**b**) weight of the mycelia. Different numbers of asterisks (*) within the panels indicate that the average of each treatment is significantly different (*p* < 0.05), *n* = 3 (15).

To analyze root colonization, chili plants were inoculated with *T. atroviride* WT, *Taswo1*-10, *Taswo1*-28 overexpressing strains, and two deletion mutants (*Taswo1*-6 Δ and *Taswo1*-8 Δ); as a control, non-inoculated plants were used. Statistical analysis of fresh weight and dry weight of mycelia coming from these experiments indicates that growth of the mycelial mat coming from plants inoculated with the WT, *Taswo1*-10, and *Taswo1*-28 strains was significantly different (Figure 2). This method to measure root colonization proved to be reliable and quantitative since it was very reproducible according to the statistical analysis performed. The control plate (non-inoculated plants) showed no fungal growth indicating that surface sterilization was effective. These results indicate that chili plant roots were colonized more effectively by the *T. atroviride* overexpressing strains than by WT strain.

When these strains were inoculated *in S. lycopersicum*, similar results were observed. The overexpressing strain *Taswo1*-28 was more effective in colonizing tomato plants than its counterparts. Again, the control without inoculation showed no mycelial growth (data not shown).

Curiously, the mutant strains *Taswo1*-6Δ and *Taswo1*-8Δ colonized the plants in a similar manner to the WT, in accordance with the plants’ phenotypes (Figure 1), suggesting again that SWOLLENIN is not essential for root colonization and that a compensatory mechanism for root colonization must exist.

Plant root colonization by *T. atroviride* was also monitored by light microscopy (Figure S5). This experiment showed that root hairs in chili plants colonized by the *Taswo1*-28 strain were longer than those in roots colonized with the WT or in non-inoculated plants (Figure S5).

### 2.3. Overexpressing and Mutant Strains Provide Protection against Fungal Infection to a Higher Extent Than the WT

**Figure 3:**
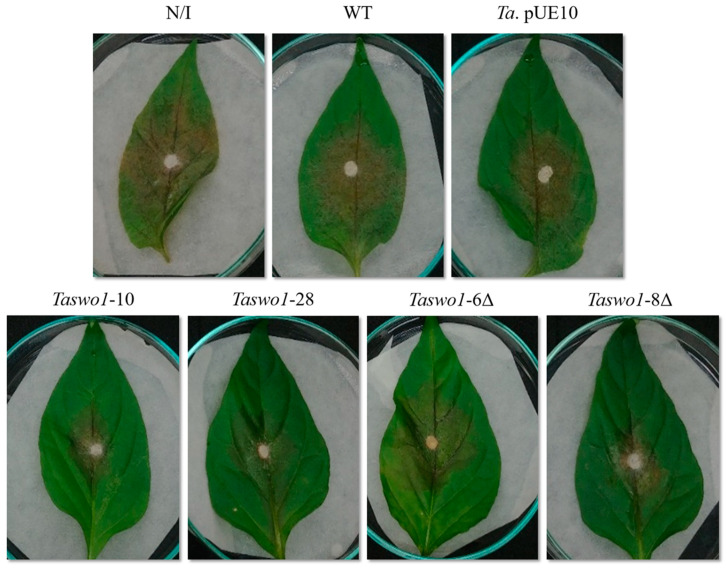
Representative image of infection resistance to *R. solani* on leaves of *C. annuum*. The upper panel shows the controls: non-inoculated plants; inoculated with the WT or the pUE10 transformant. Lower panel: leaves from plants inoculated with *T. atroviride Taswo1*-28, *Taswo1*-10; *Taswo1*-6Δ, and *Taswo1*-8Δ. The area of damage was measured using the Image J program.

**Figure 4:**
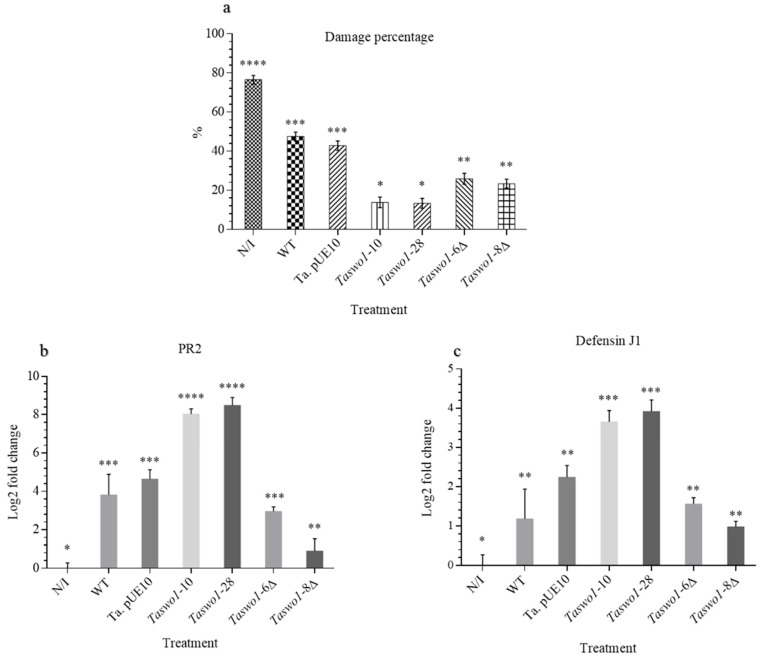
Relation between damage in leaves by *R. solani* and expression of defense genes in response to inoculation of different *Trichoderma* strains. NI, non-inoculated; WT, WT *T. atroviride*; *Taswo1*-28 and *Taswo1*-10, overexpressing strains; *Taswo1*-6Δ and *Taswo1*-8Δ, mutant strains. (**a**) analyzes of the damaged area in leaves of *C. annuum* plants treated with different *T. atroviride* variants. N/I without inoculation. (**b**) qRT-PCR relative expression of PR2 expression. (**c**) qRT-PCR relative expression of defensin J1. Ribosomal protein L13e and DNA polymerase family B were used as constitutive controls. Anova variance tests were performed in all cases. Different numbers of asterisks (*) within the panels indicate that the average of each treatment is significantly different (*p* < 0.05), *n* = 3 (15).

Since we observed a clear effect of the overexpressing strains in growth promotion and root colonization, we decided to explore if the overexpression of SWOLLENIN could also confer enhanced resistance to the infection by fungal pathogens. Leaves of *C. annuum* plants treated with the different sets of *T. atroviride* strains were inoculated with *Rhizoctonia solani* or *Alternaria solani*. Infected leaves showed different kinds of lesions according to the phytopathogen used: *A. solani* grew as mycelium on the surface of the leaves, while *R. solani* caused chlorotic spots on the leaves. A representative example is shown in Figure 3. Non-pathogen-treated leaves (control for asepsis) showed no microbial growth or lesions of any kind. Leaves of plants without *T. atroviride* inoculation were almost completely invaded by the phytopathogenic fungi (around 80% of the leaf area was affected (Figure 3 and Figure 4)). The area of the lesions caused by the fungi on leaves of plants inoculated with the overexpressing or mutant strains were much smaller (around 10% and 30%, respectively) than when inoculated with the WT strain (around 50%, Figure 3 and Figure 4).

Interestingly, both mutant strains protected the leaves to some extension performing better than the WT strain (although significantly lesser than the overexpressing strains), indicating that other defense mechanism(s) independent of SWOLLENIN must be triggered by the plants when the fungus lacks SWO1 (Figure 4a; see Discussion).

To determine the impact of the different strains on the plant defense response, we determined the expression of two marker genes, a defensin gene to monitor induced systemic response (ISR) and a pathogenesis-related protein (PR) for systemic acquired resistance (SAR). RT-qPCR experiments showed that the expression of both marker genes was strongly stimulated by the *Taswo1*-28 and *Taswo1*-10 *Trichoderma* strains, while their expression in plants inoculated with the WT strain was also higher than those observed in non-inoculated plants (Figure 4b,c). Interestingly, the mutant strains expressed both the PR gene and the defensin gene in a similar way as the WT, confirming the notion that other mechanisms, independent of the SAR and ISR responses, must play a role in the plant defense response when SWOLLENIN is absent (see Discussion). It is worth noting that although strain *Taswo1*-8Δ shows a bit lower PR expression when compared to *Taswo1*-6D, it still expresses this protein at higher levels than the non-inoculated plants.

### 2.4. Overexpression of Taswo1 Provides Abiotic Stress Protection in C. annuum Plants

*Trichoderma* abilities to alleviate abiotic stresses are known. To explore if better root colonization by *Trichoderma* would enhance abiotic stress responses, three-week *C. annuum* plants were inoculated with the WT *Trichoderma* strain or with *Taswo1*-28 and 24 h later subjected to cold stress (4 °C for 6 h) and its survival evaluated after 30 days in standard conditions (28 °C). The result shows that 100% of the plants inoculated with the *Taswo1*-28 strain survived this cold stress treatment, while only 50% of the plants inoculated with the WT strain survived. None of the control plants (non-inoculated) survived the cold stress treatment (data not shown).

In a similar way, plants inoculated with the WT strain, the overexpressing and mutant strains, and non-inoculated plants were subjected to heat stress treatment (40 °C for 6 h). A total of 100% of the plants inoculated with *Taswo1*-28 survived when returned to standard conditions for 30 days. Comparatively, only 30% of the plants survived when inoculated with the WT strain, while all of the non-inoculated plants were dead after 30 days (data not shown).

**Figure 5:**
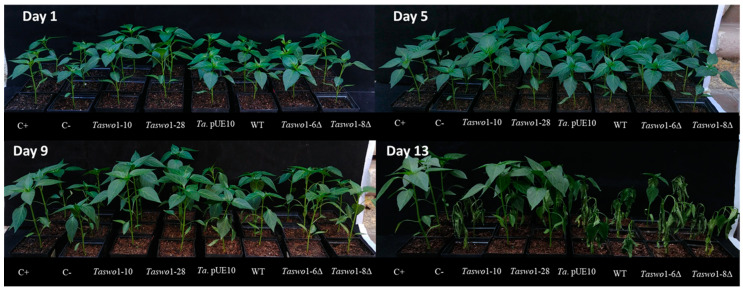
Water stress effects on *C. annuum* plants inoculated with different *T. atroviride* strains. Plants were grown for 30 days under a normal water regime, and at this time, irrigation was suspended for thirteen days. Then, irrigation was resumed, and 3 days later, the survival rate was assessed. C+ regularly watered plants without inoculum. C- non-inoculated, non-irrigated plants. WT: wild type *T. atroviride*; pUE10 *T. atroviride* with the empty vector, *Taswo1*-28, and *Taswo1*-10: overexpressing strains; *Taswo1*-6Δ and *Taswo1*-8Δ: mutant strains for *Taswo1*. Experiments were performed in triplicate.

A similar result was obtained when plants inoculated with the different *T. atroviride* strains were subjected to drought stress. The plants were deprived of water for up to 13 days, when damage was evident, and then re-watered again to assess survival. Figure 5 shows representative images of this experiment taken three days after resuming irrigation.

The survival rate for the different treatments was assessed after three days of re-watering. A positive control of normally irrigated plants was added to compare against the treatments. A total of 100% of the plants survived this treatment when inoculated with the SWOLLENIN overexpressing strains. Interestingly, neither the WT nor the mutant strains helped the plant withstand this treatment.

### 2.5. Osmolyte Accumulation under Drought Conditions in Plants Inoculated with Overexpressing and Mutant Strains

**Figure 6:**
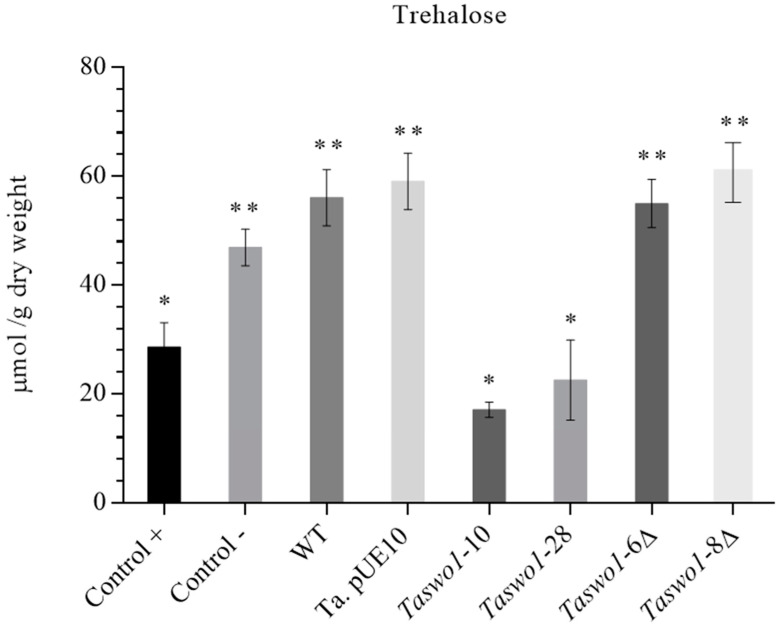
Trehalose contents in leaves of plants treated with different *Trichoderma* strains. Control+: irrigated but non-inoculated plants; control-: non-inoculated and non-irrigated plants. WT: wild type *T. atroviride*; pUE10 *T. atroviride* with the empty vector, *Taswo1*-28, and *Taswo1*-10: overexpressing strains; *Taswo1*-6Δ and *Taswo1*-8Δ: mutant strains for *Taswo1*. Different numbers of asterisks (*) within the panels indicate that the average of each treatment is significantly different (*p* < 0.05).

Osmolyte accumulation is one of the mechanisms plants use to deal with low water activity. To assess if this mechanism was induced by the overexpression of SWO1 by *Trichoderma*, plants were inoculated with different strains and subjected to water stress. Curiously, trehalose was found in lower concentrations in plants inoculated with the overexpressing strains compared to the rest of the strains, which showed higher levels than the uninoculated control (Figure 6). Erythritol and mannitol were also produced more or less in the same amounts, but no significant difference between the treatments was observed (data not shown), with the exception of those plants that were normally irrigated.

**Figure 7:**
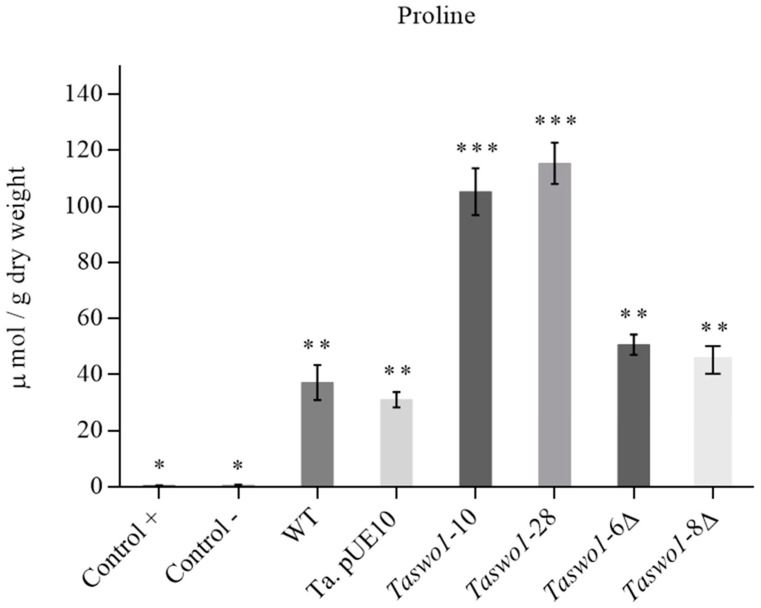
Proline contents in leaves of *C.* inoculated with different strains of *T. atroviride*. C+: irrigated non-inoculated plants; C-: non-inoculated and non-irrigated plants; WT: wild type *T. atroviride*; pUE10 *T. atroviride* with the empty vector; *Taswo1*-28 and *Taswo1*-10: overexpressing strains; *Taswo1*-6Δ and *Taswo1*-8Δ: mutant strains for *Taswo1*, none of the inoculated plants were irrigated for 13 days. Different numbers of asterisks (*) within the panels indicate that the average of each treatment is significantly different (*p* < 0.05).

However, proline was accumulated strongly in the plants inoculated with the overexpressing strains when compared to plants inoculated with either the WT or the mutant strains (Figure 7).

## 3. Discussion

*Trichoderma* species are avirulent opportunistic plant symbionts able to colonize plant roots and produce compounds that stimulate growth and plant defense mechanisms^4^. SWOLLENIN is a protein with expansin-like activity that was identified in *T. reesei* and has been related to the fungal cellulolytic activity during its saprophytic growth. On a first instance, it was proven that it could disrupt the structure of cotton fibers without detectable formation of reducing sugars [27], but afterward, its hydrolytic activity was demonstrated [33]. An important barrier encountered by *Trichoderma* during root colonization is the plant cell wall, which is comprised of a complex matrix of polysaccharides, being cellulose one of the most abundant. Due to the activity of SWOLLENIN and a previous report by Brotman et al. [3], we proposed that overexpression of SWOLLENIN in *T. atroviride* would enhance its root colonization abilities, as shown for *T. asperellum*. However, the work by Brotman et al. [3] was performed mostly in vitro, and their results regarding biotic resistance were performed with just a synthetic CBM of SWOLLENIN, while our work measures the effect on the whole adult plant, and the complete *TaSWO1* gene was expressed. Here, we show that overexpression of SWOLLENIN in strains *Taswo1*-10 and *Taswo1*-28 indeed enhanced root colonization of chili plants when compared with the WT strain of *T. atroviride*. While the work by Brotman et al. [3] also proved an increase in root colonization when overexpressing the *TasSWO1* gene, we observed in addition that this phenomenon led to higher biomass production of the shoot and root of the plants inoculated with the SWOLLENIN overexpressing strains. This fact also allowed us to describe the behavior of plants inoculated with *Taswo1* deletion mutants, which behave very similar to the WT strain, indicating that SWOLLENIN is not essential for root colonization and that there must be compensatory mechanisms for this and other traits (see below).

Moreover, the strains tested in this study were not able to cause significant effects on plant biomass augmentation in *P. vulgaris* plants, indicating a certain level of specificity. The latter belongs to sub-class: Rosidae, while cucumber, chili, and tomato share the same class: Magnoliopsida, but belong to different subclasses: Dilleniidae (cucumber) and Asteridae (chili and tomato, that are in the same family: Solanaceae.). It is generally accepted that there is no or little plant specificity regarding *Trichoderma* spp. ability to colonize plants. However, in a study conducted by Cripps-Guazzone (2014) [34], it was shown that competition in the rhizosphere between different *Trichoderma* strains “showed that the relationship between *Trichoderma* and the plant is dependent on the *Trichoderma* isolate and the plant species”. As an example, from several isolated *Trichoderma* spp., one of the least competent species was the only one able to colonize onion roots. In another work, soil fungal communities were isolated from oilseed rape and strawberry rhizosphere. It was found that although *Trichoderma* spp. showed great diversity in both cases, BOX-PCR fingerprint experiments indicated a large degree in plant specificity [35]. The specificity mechanisms of *Trichoderma*-plant interactions have been scarcely studied because, as stated before, it is considered that no specific barriers impede plant colonization. However, the role of certain proteins could provide a specificity barrier in the interaction of *Trichoderma* with the plant cell wall. Among them are the CBM of proteins such as SWOLLENIN, cerato-platanins, and lytic enzymes as glucanases and cellulases, which are classified in 55 families and differ in their substrate specificity [36]. Other proteins involved in plant colonization by *Trichoderma* are hydrophobins, which have been found to be species-specific [37] and usually have several members in filamentous fungi [38]. Furthermore, impairment of a *T. asperellum* hydrophobin gene prevented the attachment of the fungus to cucumber roots [39], and hydrophobins have also been linked to plant attachment and root colonization in *Trichoderma* spp. in a study that identified effector proteins [25]. Finally, plant lectins have been shown to play a crucial role in the specificity in other symbiotic systems such as that of *Rhizobium* with legumes [40]. Lectins in *Trichoderma* spp. have been widely studied and shown to play an important role in mycoparasitism [41], but to the best of our knowledge, no studies of *Trichoderma* lectin interactions with plants have been performed, which could be another barrier of specificity.

Because it is well documented that colonization by *Trichoderma* increases plant defense responses [4,42,43], we also analyzed the enhanced resistance to phytopathogenic fungi induced by overexpressing strains. In this work, we present data showing that SWOLLENIN is a suitable elicitor of the plant defense responses since we found significantly increased resistance toward the phytopathogenic fungi *R. solani* or *A. solani* in chili plants colonized by the overexpressing strains compared with the WT strain. Many *Trichoderma* spp. are able to induce systemic changes in plants, which are frequently related to increased levels of PR proteins and the accumulation of phytoalexins [44]. The application of a synthetic version of the CBM of SWOLLENIN of *T. asperellum* reduced the disease provoked by *Pseudomonas* and *Botrytis* in cucumber plants [3]. In this regard, the production of antifungal compounds in leaves of plants colonized by *Trichoderma* has been shown [45] It has been reported that inoculation of a strain of *T. harzianum* on bean roots reduced considerably the lesion area provoked by *B. cinereal* [46] It has also been shown that *T. atroviride* can enhance systemic disease resistance in *Arabidopsis thaliana* through jasmonic acid/ethylene and salicylic acid pathways [15].

Here we show that disease severity caused by *R. solani* or *A. solani* on *Capsicum* leaves was significantly reduced in plants treated with an overexpressing strain compared with the WT or untreated plants. Together with the defense gene expression experiments, these results suggest that overexpression of SWO1 in *T. atroviride* results in enhanced activation of the JA/ET and SA pathways, which consequently suppresses pathogen infection in *C. annuum*. Interestingly, the mutant strains in *swo*1 also conferred a suitable level of protection, performing even better than the WT (see below).

The defense response in plants is complex and involves many steps, signal transduction pathways, transcription factors, and protein and metabolite production. Several studies have indicated that root colonization by *Trichoderma* strains results in increased levels of defense-related enzymes in plants, including peroxidases, chitinases, and β-1-3-glucanases [4,43,47]. In our study, we found that both PR2 and defensin J1 encoding genes were up-regulated in leaves of plants inoculated by strains *Taswo1*-10 and *Taswo1*-28 when compared to plants inoculated with the WT strain or to untreated plants. Mutant strains showed a level of expression similar to the WT for both genes. These results suggest that enhanced expression of PR proteins in plants inoculated with *T. atroviride* SWO1 overexpressing strains could be involved in the systemic response to suppress diseases in *Capsicum* since the WT and the mutant strains showed lower levels of expression. Defensin was also overexpressed in plants inoculated with the SWO1 overexpressing strains, and it has been shown to effectively inhibit the growth of pathogenic microorganisms and also generate tolerance to abiotic stress conditions in plants [48].

The phenotype observed in plants inoculated with the mutant strains (i.e., better protection than the WT, but similar expression of the PR and defensin genes) is still to be explored carefully. It has been reported that cerato-platanins of *Trichoderma* are major elicitors of plant defense responses [23,24]. Cerato-platanins are related to expansins, SWOLLENINs, and loosenins, regarding its amorphogenic activity [49], although their role is still in debate [50,51]. The observed phenotype in plants inoculated with the mutant strains could be due to the overexpression of cerato-platanins, for which a role in root colonization and elicitation of defense mechanisms [24] has been demonstrated in several *Trichoderma*-plant interactions [50,51,52]. In support of this idea, the work by Crutcher et al. [53] showed that in *sm2* mutants (a cerato-platanin paralog), SM1 (another cerato-platanin) was spontaneously overexpressed. So, it could be possible that in the *swo1* mutants, cerato-platanins are overexpressed, conferring to these strains a similar phenotype to that of the WT regarding root colonization, biomass enhancement, and drought tolerance. It is worth noticing also that the mutants’ performance to avoid fungal infection better than the WT could be related to other defense mechanisms different from ISR and SAR or to an earlier response in mutant-inoculated plants. Among these, calcium signaling could be involved, since it has been shown that plants are capable of distinguishing signals originated by different fungal partners [54]. Furthermore, it has been shown that a hydrophobin from *T. longibrachiatum* provokes a rapid increase in Ca^+2^ in *Lotus japonicus* cells [55]. In addition, production of reactive oxygen species is also one of the first steps in response to pathogen infection, before the ISR or SAR systems are fully functional. Finally, a SAR and ISR independent induced defense system that relies in a F-BOX protein (SON1), has been shown in *A. thaliana* [56]. It has also been shown that another F-BOX protein confers resistance to powdery mildew in *Vitis pseudoreticulata* [57]. Further analyzes will be performed to explore the plant defense mechanisms in the deletion mutant-inoculated plants.

We also showed that at least part of the mechanism of drought tolerance is due to proline accumulation. Curiously, other compatible solutes were found in lesser amounts in plants inoculated with overexpressing strains (specially trehalose). This could be because carbon metabolism in the overexpressing inoculated plants is funneled to produce a more efficient osmolyte such as proline. In addition, the strategy to withstand drought seemed to be different when plants were inoculated with the overexpressing strains since plants inoculated with the non-overexpressing strains accumulated trehalose, while a different osmolyte (proline) was found in the plants inoculated with the overexpressing strains. This novel observation deserves and will require more experimentation to unravel this phenomenon.

We suggest that SWOLLENIN could be causing increased amorphogenesis in the cell wall of plants treated with the overexpressing strains, and this facilitates the fungus to colonize the root, activating the plant signaling pathways in a more extensive manner than that in the control plants. It is also a possibility that enhanced amorphogenesis of the cell wall could liberate oligosaccharides (either by lytic enzymes produced by the fungus or by SWOLLENIN itself, according to the report of Andeberg et al. [33]), which could be responsible for a stronger activation of the defense and stress resistance pathways. This study contributes important insights for understanding the nature of beneficial *Trichoderma*-plant interactions and may be used to improve *Trichoderma* biocontrol and biofertilization abilities.

## 4. Materials and Methods

Strains and Growth Conditions. For propagation and maintenance, *T. atroviride* strains IMI206040 WT, *Taswo1*-10, *Taswo1*-28, *Taswo1*-6Δ, *Taswo1*-8Δ, and a transformant with the empty vector (pUE10 [32]), were grown on solid PDA medium (2% potato, 2% dextrose, and 1.5% agar) for 5 days at 28 °C. The spores were then harvested and resuspended in sterile distilled water up to a concentration of 10^8^/mL and stored at 4 °C until further use. For the transformant strains, 100 µg/mL of hygromycin were supplemented.

Plant Material. Chili (*Capsicum annuum*) seedlings were surface disinfected as described by Glazebrook and Weigel [58]. Seeds were sown in Murashige and Skoog medium (1X MS) supplemented with 3% sucrose (Bioxon) and gelled with 0.7% agar phytagel (Sigma-Aldrich). After germination of seeds, three-week-old plantlets were transferred to plastic pots filled with Metro mix in a controlled environment: 24–28 °C, 75% relative humidity, and a circadian cycle of 18 h light/6 h darkness, the intensity of light 100 µmol m^−2^s^−1^ for three weeks when they reached an approximate height of 3 cm on average. *Solanum lycopersicum* (CID F1 saladett variety) and common bean (*Phaseolus vulgaris* L. bv. Negro Jamapa) were subjected to the same procedure as for chili to perform the interaction tests.

Construction of *T. atroviride* Strains Overexpressing the *TaSWO1* Gene or Deletion Mutant Strains. The native SWOLLENIN (MycoCosm ID80187) cDNA from *T. atroviride* was amplified by PCR using as a template cDNA obtained from *T. atroviride* in confrontation with *Rhizoctonia solani* AG5 before the fungi made contact, as reported by Reithner et al. [31]. Primers swoF (5′ *GCGGCCGC*atcATGTTGCGTAAACTTAGCCTAC 3′) containing a *Not*I restriction site (italics) and swoR (5′ *GAATTC*CAGTTCTTACTAAACTGTAGAGC 3′) containing an *Eco*RI restriction site (italics) were used to amplify the PCR product for further subcloning into the site-directed integrative vector pUE10 under the *PKI* promoter of *T. reesei* [32] giving rise to pUE10::*Taswo1*. The PCR conditions were: 94° C 5′, 35 cycles of 94 °C 40″, 58 °C 30″ and 72 °C 90″ and one cycle of 10′ at 72 °C. Several clones were sequenced by the method of Sanger to verify they were correct. Plasmid pUE10::*Taswo1* was transformed into the WT *T. atroviride* strains, and hygromycin-resistant colonies were selected. At least three monosporic passes were performed to ensure genetic homogeneity.

*SWO1* Mutant Design. The *SWO1* gene was replaced by a hygromycin B resistance-conferring cassette using the double-joint PCR method. Eight pairs of primers were designed for the replacement construct: R1SwoFwd (CCCTTGAGAGAGAGCAGGTGTTTGTCC) and R1SwoRev (CCTTCAATATATCAGTTAACGTCGATCGGTGCCTCCTGTCAAGAGAGATTTTTGG) were used to amplify the 5′ flanking region of *SWO1*, R2SwoFwd (CACTCGTCCGAGGGAGGGCAAAGGAATAGGCTGCTGGGTGTGATTCCACACAAGGATCG), and R2SwoRev (GTTTATCACCAGTCTCGAACACACAGAGAGAGC) were used to amplify the 3′ flanking region of *SWO1*, hph-F (GATCGACGTTAACTGATATTGAAGGAG) and hph-R (CTATTCCTTTGCCCTCGGACGAGTG) were used to amplify the *HPH* gene, Nestswo1 (GGGTCAGATGGATGGCTTCCAAAGTGTGTG) and Nestswo-2 (ATTGAATAGAGAGAGGAGTTTATCACCAGTCTCG) were used to amplify the full replacement construct. The PCR conditions to amplify the 5′ flanking region, 3′ flanking region of *SWO1,* and the *HPH* gene were as follows: 94 °C 3′, 35 cycles of 94 °C 30″, 58 °C 30″ and 72 °C 90″ and one cycle of 10′ at 72 °C. The conditions to fusion 5′ flanking region, 3′ flanking region and the *HPH* gene were: 94 °C 3′, 10 cycles of 94 °C 30″, 58 °C 60″ and 72 °C 10′ and one cycle of 20′ at 72 °C. The PCR conditions to amplify the full replacement construct were: 94 °C 3′, 35 cycles of 94 °C 30″, 58 °C 60″ and 72 °C 5′ and one cycle of 10′ at 72 °C. The resulting product was transformed into the *T. atroviride* WT strain, and hygromycin-resistant colonies were selected. At least three monosporic passes were performed to ensure genetic homogeneity.

Transformation and Monosporic Selection. Transformation was carried out according to Herrera-Estrella et al. [59]. The transformed cells were plated on PDA plates containing hygromycin B (100 µg/mL) in the presence of light until spores were obtained. These spores were diluted and re-plated at least three times until stable lines were obtained. Several stable transformant strains were characterized. All of them showed similar behavior, so we chose *Taswo1*-10 and *Taswo1*-28 for further experiments. The insertion of pUE10::*Taswo1* in the *T. atroviride* strains was verified by PCR using genomic DNA as a template and oligonucleotides swo1Fwd (5′ CGCAAGAACGCTATGGTAGCT GG 3′) and blu17R (5′ AGTGTGGAGTTGGTCAAATGATGGG 3′), since only this combination would amplify a 1500 bp product if the *Taswo1* gene is integrated at the BLU17 locus (not shown). In addition, this result was confirmed with a Southern blot analysis for strain *Taswo1*-28 (Figure S1). The expression of *Taswo1* was verified by RT-PCR using cDNA obtained from cultures of the WT strain as a negative control, and all the genetically modified strains tested in this work were grown in PDA liquid medium (Figure S2) and performing the PCR with oligos swoF and swoR. Three monosporic cultures were performed to obtain the spores used for inoculation.

Growth rate of the *T. atroviride* strains Fresh PDA plates with the different strains were grown to confluence, and using a boring stopper, 7 mm agar disks were extracted and used as inocula into fresh PDA plates to monitor growth rate. They were examined daily, and radial growth was measured to determine their growth speed in cm per day.

Inoculation of Chili Plants with the Mutant or WT Strains of *T. atroviride*. For the pot experiments, three cm high, chili plants were inoculated with 1 × 10^8^ spores of the WT strain the *Taswo1*-10, *Taswo1*-28, *Taswo1*-6Δ, and *Taswo1*-8Δ strains or the empty vector pUE10, non-inoculated plants were used as control.

Plant Growth and Root Colonization Comparison. For each fungal strain, seven plants in every one of three lots were inoculated, as explained above. Every fifteen days, plants were observed and compared on the basis of the following parameters: shoot length of the plant, leaf area (leaves were taken from the same positions in the shoots to make the comparisons), at day 30, dry weight of plant root and shoot were measured.

Root Colonization Assay. A novel, cheap method to measure root colonization was implemented for root colonization assays. This method avoids the use of expensive equipment and reagents and also the participation of highly trained personnel (such as RT-qPCR). Roots were detached from different plants after 20 days post-inoculation with *Taswo1*-10 or *Taswo1*-28 or WT or non-inoculated plants and then extensively washed with water. After surface sterilization in 2% NaOCl for 2 min, the roots were washed abundantly with sterile distilled water. In all samples, portions of one cm in length were cut below the root base in equivalent positions for all samples (as measured in cm); the samples were weighed to ensure the same biomass amount for all samples and were homogenized using a pestle and mortar and diluted 10^3^ times in sterile water. Serial dilutions were inoculated on Petri dishes of PDA, which contained a cellophane membrane. After 48 h, the cellophane membrane was lifted from each Petri plate so that the complete mycelial mat was considered to determine fresh and dry weight. Three biological replicas with 5 plants each were examined using quantitative analysis. The rationale for this method is that if the root has been better colonized and has more hyphae, the inoculum on the Petri dishes should be more abundant, and a greater growth should be expected, which proved to be the case.

Microscopy. Plants inoculated with WT or *Taswo1*-28, and non-inoculated (control) plants were harvested from the medium, and excess agar was removed. Roots collected were chopped into 1 cm-long pieces and transferred into vials. Roots were treated by adding 10% KOH after rinsing with ethanol. The samples with KOH were transferred into a water bath at a temperature of 80 °C for 40 min. The KOH solution was rinsed from the root, and 3% hydrogen peroxide was added for 3 min. After this treatment, a staining solution containing 0.5 g of methylene blue, 400 mL of lactic acid, 400 mL of glycerol, and 200 mL of distilled water was added to root samples and kept in a water bath for 2 min. The stained roots were distained using a 50% glycerol solution. For each strain, at least twenty 1 cm stained roots were mounted on a slide and observed under a compound microscope at 100X magnification. Fifteen root hairs were measured in each sample.

RNA Extraction and Gene Expression Analysis by RT-qPCR. Total RNA from fungal mycelium was extracted according to Viterbo et al. [60]. Expression levels of defensin J1-2F (gb|EU560903.1) and PR (gb|AF227953.1|AF227953) were measured by quantitative reverse transcription-PCR (RT-qPCR) using primers: J1-Fwd: GCACACTCCATGCGTTTCTT; J1-Rv: CGCAAGTTCTTGCCTCAACA for DEFENSIN J1 and PR2-F (5′ GTT AGG TCG TTC ATT GAT CCG ATT A-3′); PR2-R (5′ AGT GAA CCA TCT TGT ACC ACC AC-3′) for PR2. A fragment of the elongation factor E1 was used as a standardization control (primers: FE-Fwd TCCAACCCTTCTTGAGGCTC; FE-Rv CTGTCCCTGTTGGTCGTGTA). To eliminate genomic DNA, RNA was treated with DNaseI (Thermo Fisher Inc.) according to the manufacturer’s instructions, and its concentration and purity were measured by the ratio of absorbance at 260 and 280 nm. The cDNA was synthesized using 500 ng of each DNase-treated RNA and 10 pmol of the specific reverse primer with a cDNA synthesis kit (Revert Aid H First Strand kit; Thermo Fisher Inc.). The cDNA obtained was used as the template for real-time PCR (RT-qPCR) assays. DNA polymerase family B (Triat2 ID: 53190) and ribosomal protein L13e (Triat2 ID:257690) [61] were used as an internal control in the same samples to normalize the results obtained. All RT-qPCRs were performed in triplicate for each gene of each strain. The quantification *total of 40 cycle* technique used to analyze the data was the 2^−ΔΔ*CT*^ method reported by Livak and Schmittgen [62]. The reproducibility of the whole procedure was determined by performing cDNA synthesis and RT-qPCR experiments with three separate RNAs extracted from each strain. Similar results were obtained for the transcription of measured genes in the repetitions and with the internal control (housekeeping) used for normalization. The cDNA obtained was used as a template for real-time PCR was performed with the I Cycler 480. Amplification conditions were 10 min at 95 °C, and two step cycles 95 °C for 15 s and 60 °C for 40 s.

Infection with Pathogenic Fungi. Chili plants were grown in soil in a controlled environment (24–28 °C, 75% relative humidity, and a cycle of 18 h light/6 h darkness). Plants were inoculated with the WT or each of the genetically modified strains, and control plants without inoculation were taken for the experiment. Leaves from several plants were detached from the same position (node) in each plant and were placed in Petri plates on wet filter paper to maintain high humidity, and the plates were wrapped in plastic bags. Two detached leaves from each of three different plants per treatment were inoculated with 5-mm-diameter mycelial agar disks (as described above for the growth rate experiments) of *Rhizoctonia* *solani* AG5, or *Alternaria solani* (IMI381020, both from the LANGEBIO-CINVESTAV collection) strains taken from 7-day-old cultures grown on PDA. The disks were placed in the middle of each leave [63]. After 24 h, the disks were removed, and disease development was assessed 3 d after inoculation. This analysis was performed at room temperature during the entire experiment. To calculate the amount of inhibition, we used the ImageJ program to measure the total leaf area and the area invaded by the fungus (for *A. solani*) or the chlorotic regions in the leaf (for *R. solani*). Then we divided the affected area by the total area to obtain the percent of damage caused by the phytopathogens.

Cold Stress Treatment. Plants were grown in a controlled growth chamber. The plants were inoculated as mentioned above with the WT or *Taswo1*-28 strains and non-inoculated plants as a control. After 24 h in controlled conditions, we conducted cold treatment at 4 °C, for 6 h and then returned the plants to controlled conditions, and the results were evaluated after 30 days.

Heat Stress Treatment. We followed the procedure in a similar way as for the cold stress experiments but then conducted a heat treatment of 40 °C for 6 h and then returned the plants in controlled conditions for 30 days.

Drought Stress Treatment. Plants were grown with a photoperiod of 16 h of light and 8 h of darkness at 27 ± 2 °C under a normal water regime for 30 days. Then, irrigation was suspended for thirteen days. After this period, irrigation was resumed, and 3 days later, the survival rate was assessed. Experiments were performed in triplicate.

Statistical Analysis. All experiments were conducted in 15–35 replicates. Data were subjected to an analysis of variance (ANOVA), which was applied to determine significant statistical differences between the different cases. Shapiro–Wilk’s W, Kolmogorov–Smirnov, and Lilliefors tests were performed to analyze the normality of the data set. For analysis of homogeneity of variance, Levene or Cochran’s C Hartley’s or Bartlett’s test was used. A post hoc analysis that defines the order of the differences found in the ANOVAs was developed. Fisher LSD tests were considered for the post hoc analyses. All statistical calculations were performed in STATISTICA-7.0.

## Figures and Tables

**Table 1:** Growth rate of the different *T. atroviride* used in this work. ANOVA tests showed no significant differences among the strains’ growth rates.

Strain	Growth Rate (cm/day)	Std Dev.
WT	1.61	±0.15
*Ta.* pUE10 (BMH-0063)	1.7	±0.01
*Taswo1*-28 (BMH-0064)	1.6	±0.05
*Taswo1*-10 (BMH-0065)	1.7	±0.1
*Taswo1*-6 (BMH-0066)	1.5	±0.1
*Taswo1*-8 (BMH-0067)	1.6	±0.09

## Data Availability

Not Applicable.

## Supplementary Materials

The following are available online at https://www.mdpi.com/article/10.3390/plants10091919/s1, Figure S1: Southern blot analysis of the integration of pUE10::*Taswo1* of strain *Taswo1*-28, Figure S2: Construction of *swo1* deletion mutants and overexpression patterns of the genetically modified strains. Figure S3: Height of *Solanum lycopersicum* plants inoculated with the WT *T. atroviride* (WT) strain or the *Taswo1*-28 overexpressing strain. Figure S4: Fresh and dry weights of *S. lycopersicum* inoculated with *Taswo1*-28 overexpressing strain. Figure S5. Root hair length in chili plants inoculated with the WT strain or *Taswo1*-28; as a control non-inoculated plant.
