# Supplementary material for: Effects on Capsicum annuum Plants Colonized with Trichoderma atroviride P. Karst Strains Genetically Modified in Taswo1, a Gene Coding for a Protein with Expansin-like Activity
Source: Plants (Basel). 2021 Sep 15;10(9):1919. doi: 10.3390/plants10091919 (PMC8468806; doi:10.3390/plants10091919)
Supplement: Supplementary file 1 [file plants-10-01919-s001.zip › Supplementary material.pdf]

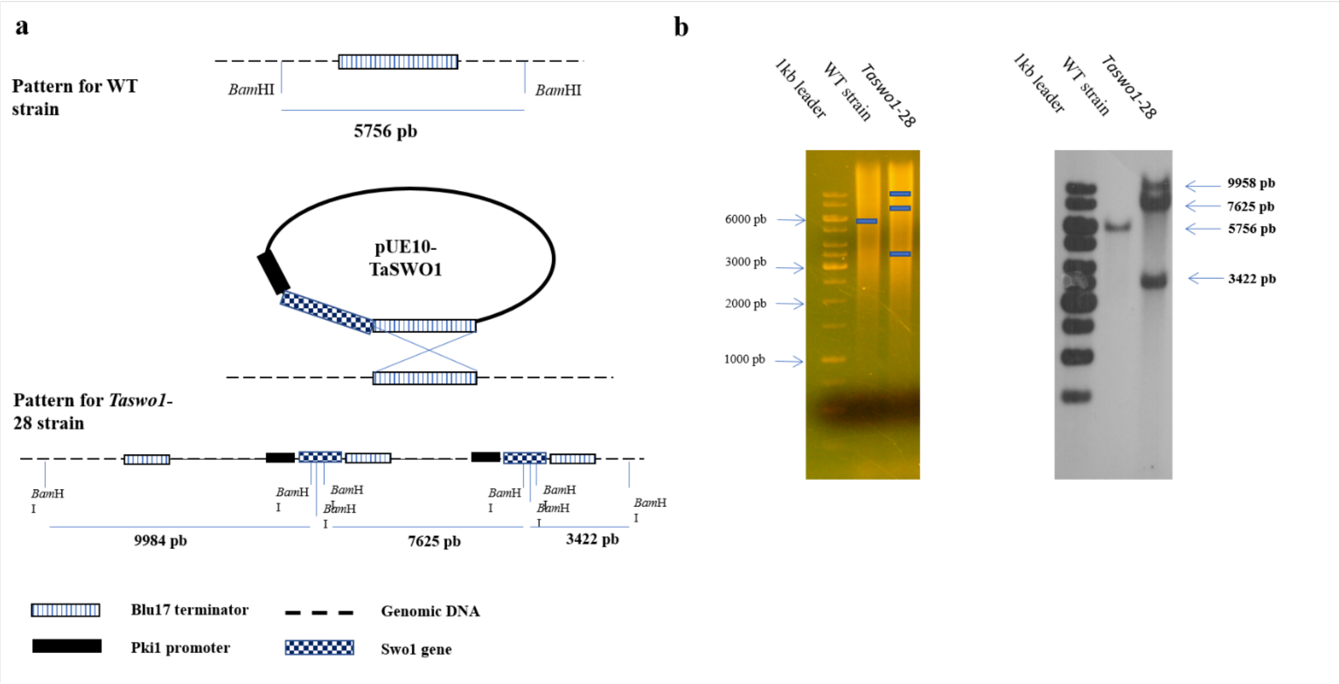

Figure S1: Southern blot analysis of the integration of pUE10::swo1 of strain *Taswo1*-28. Total DNA extracted from the wild type (WT) and from *Taswo1*-28 strains and digested with *Bam*HI. **a)** Scheme of the recombination of pUE10-*TaSWO1* vector into the *BLU17 locus* of *T. atroviride* genome. The restriction fragment pattern is explained for two integrations of vector in this región. **b)** Hybridization was performed with a ^{32}P labeled 420 bp fragment corresponding to partial *blu*17 terminator. To the left, the DNA gel stained with ethidium bromide; to the right the autoradiography after hybridization. Arrows indicate the molecular weight of the fragments.

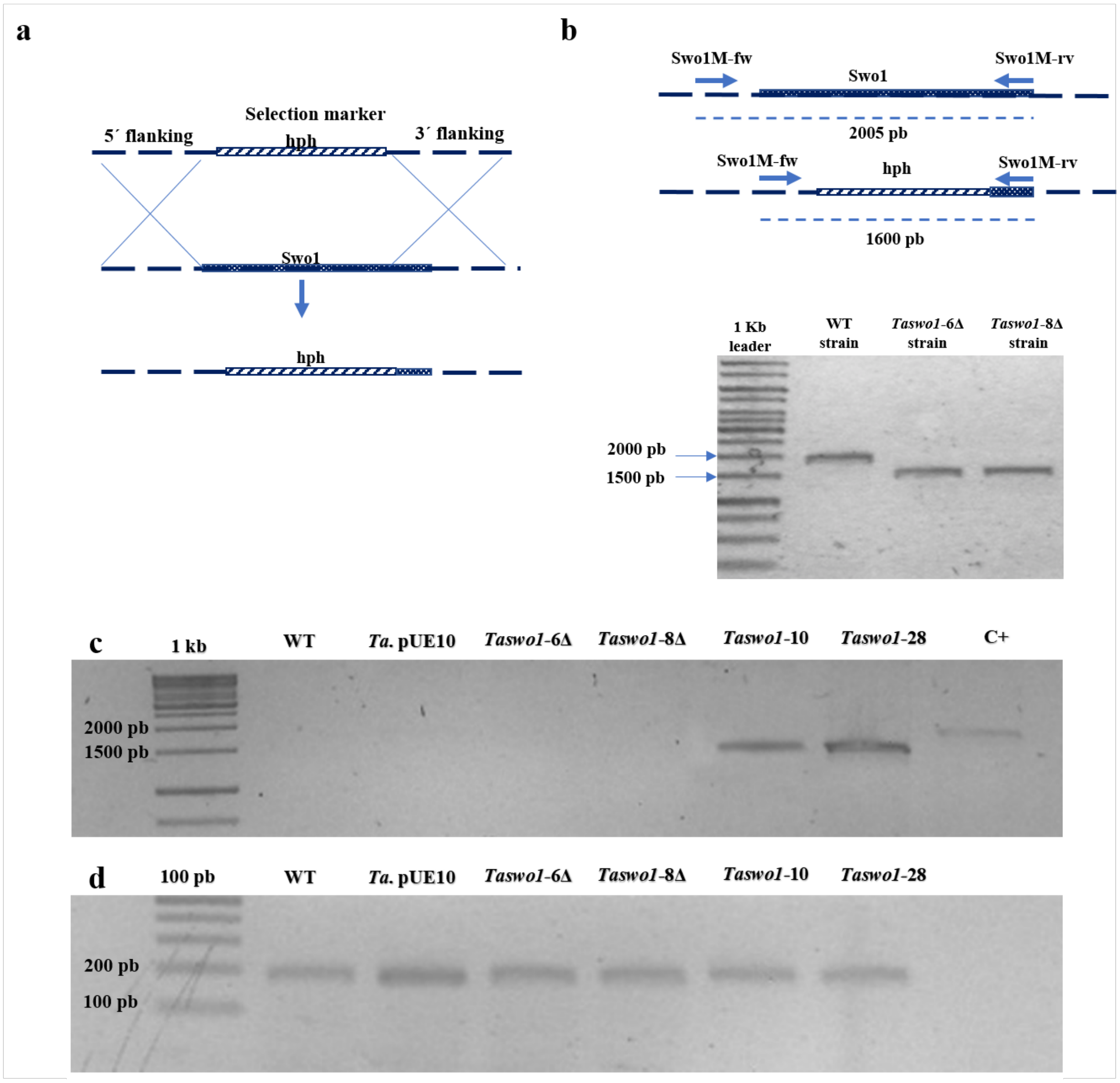

Figure S2: Construction and verification of *Taswo1* deletion mutants. **a)** Scheme of *SWO1* gene replacement by the hygromycin cassette, dotted lines represent genomic DNA. **b)** Upper part, scheme of the PCR band pattern expected for the wild type strain and the deletion mutants using primers Swo1-Mfwd and Swo-1M rev. Dotted lines represent genomic DNA, while small light blue lines represent the expected PCR product. Lower part, gel with the actual PCR products obtained using genomic DNA from each strain as template **c)** Transcriptional analysis of the RT-PCR products of WT and *TaSwo1* construct strains. RT-PCR was performed using RNA extracted from the different strains grown in PDA with hygromycin. C+ genomic DNA from the wild type was used as a positive control **d)** RT-PCR using *SWO1* primers Swo-F-n and Swo-R-n. **c)** Control using *GPD* Primers gpd-F and gpd-R

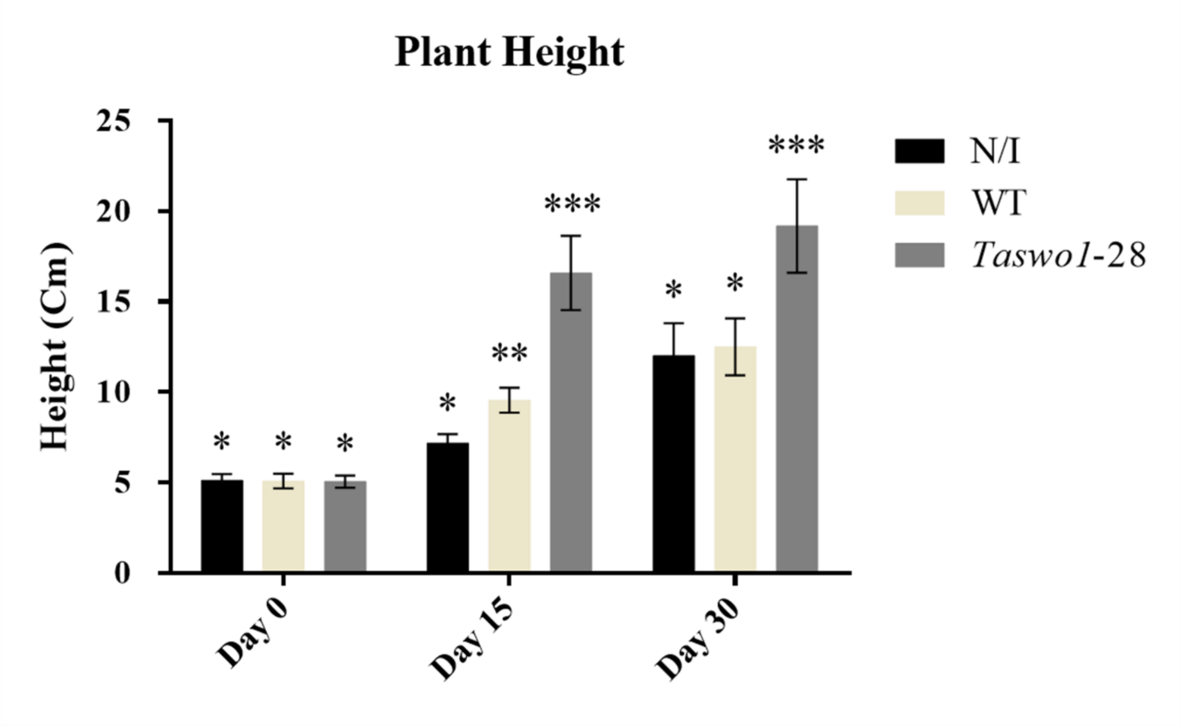

Figure S3: Height of *Solanum lycopersicum* plants inoculated with the Wild type *T. atroviride* (WT) strain or the *Taswo*1-28 overexpressing. Means of three replicas are depicted in the graph. Bars indicate standard deviation. Different number of asterisks within the panels indicate that the average of each treatment is significantly different ($p < 0.05$).

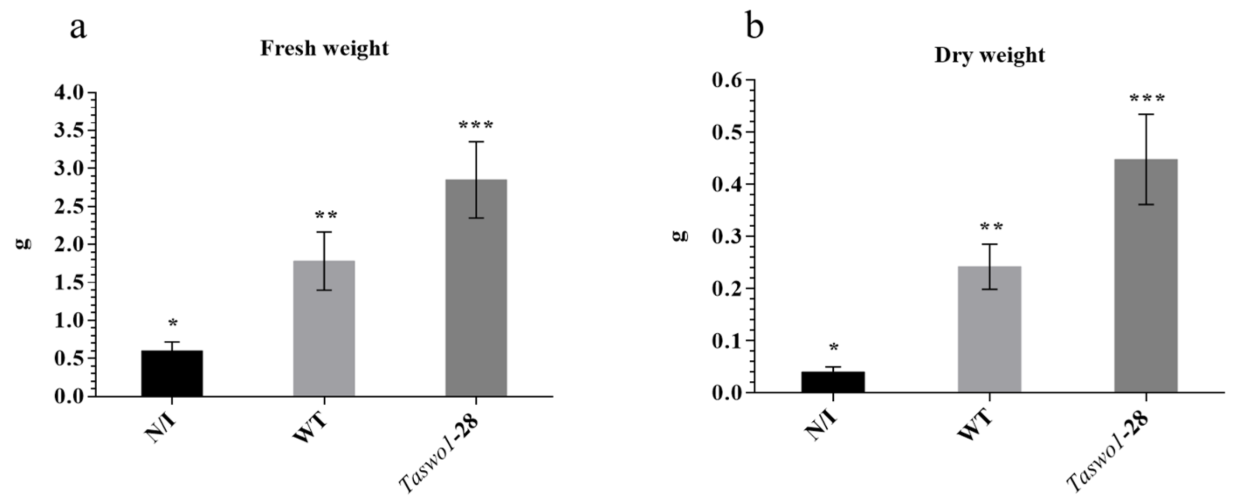

Figure S4. Biomass production of *S. lycopersicum* plants as determined by plant fresh weigth (a) and dry weights (b). Means of three replicas are depicted in the graph. Bars indicate standard deviation. Different number of asterisks within the panels indicate that the average of each treatment is significantly different ($p < 0.05$). Note that scales are different for panels a) and b) so statistics are only comparable whitin each panel.

a

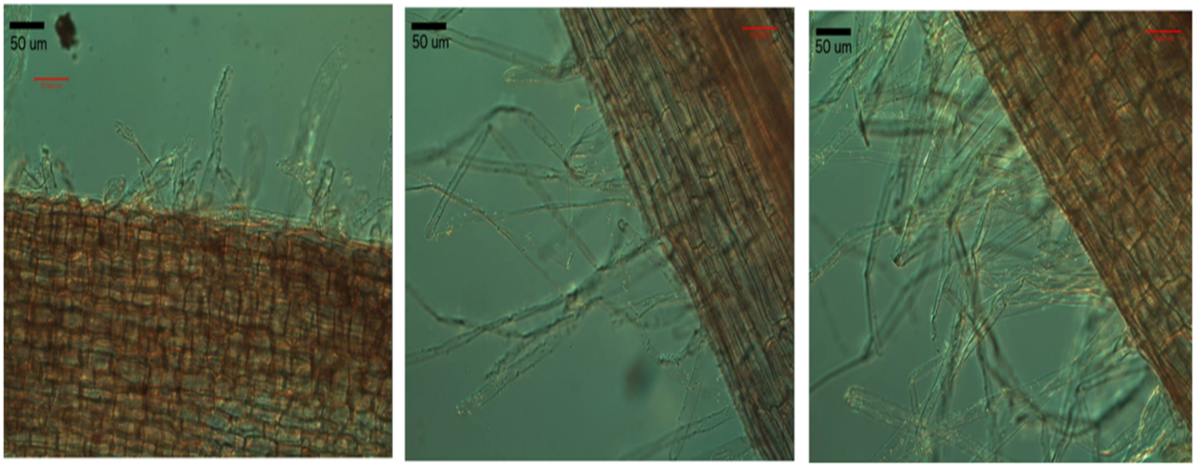

b

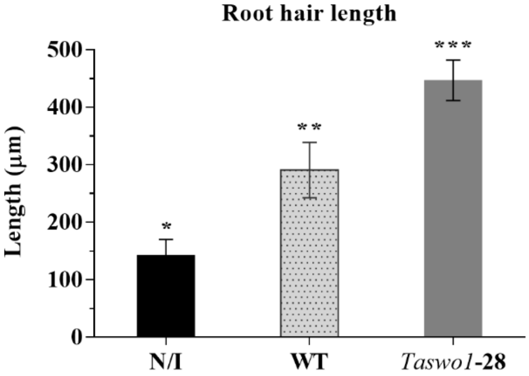

Figure S5: a) Root hair length in chili plants inoculated with the WT strain or *Taswo1*-28; as a control non-inoculated plant. b) Statistical analysis of treatments, different number of asterisks within the panels indicate that the average of each treatment is significantly different ($p < 0.05$)
